# Lack of Gut Secretory Immunoglobulin A in Memory B-Cell Dysfunction-Associated Disorders: A Possible Gut-Spleen Axis

**DOI:** 10.3389/fimmu.2019.02937

**Published:** 2020-01-08

**Authors:** Rita Carsetti, Antonio Di Sabatino, Maria Manuela Rosado, Simona Cascioli, Eva Piano Mortari, Cinzia Milito, Ola Grimsholm, Alaitz Aranburu, Ezio Giorda, Francesco Paolo Tinozzi, Federica Pulvirenti, Giuseppe Donato, Francesco Morini, Pietro Bagolan, Gino Roberto Corazza, Isabella Quinti

**Affiliations:** ^1^B Cell Pathophysiology Unit, IRCCS Bambino Gesù Children's Hospital, Rome, Italy; ^2^Diagnostic Immunology Unit, IRCCS Bambino Gesù Children's Hospital, Rome, Italy; ^3^First Department of Medicine, IRCCS San Matteo Hospital Foundation, University of Pavia, Pavia, Italy; ^4^Department of Molecular Medicine, Sapienza University, Rome, Italy; ^5^Second Department of Surgery, IRCCS San Matteo Hospital Foundation, University of Pavia, Pavia, Italy; ^6^Department of Translational and Precision Medicine, Sapienza University, Rome, Italy; ^7^Department of Medical and Surgical Neonatology, IRCCS Bambino Gesù Children's Hospital, Rome, Italy

**Keywords:** common variable immune deficiency, gut mucosal immunology, plasma cell, splenectomy, transmembrane activator and calcium-modulator and cyclophilin ligand interactor

## Abstract

**Background:** B-1a B cells and gut secretory IgA (SIgA) are absent in asplenic mice. Human immunoglobulin M (IgM) memory B cells, which are functionally equivalent to mouse B-1a B cells, are reduced after splenectomy.

**Objective:** To demonstrate whether IgM memory B cells are necessary for generating IgA-secreting plasma cells in the human gut.

**Methods:** We studied intestinal SIgA in two disorders sharing the IgM memory B cell defect, namely asplenia, and common variable immune deficiency (CVID).

**Results:** Splenectomy was associated with reduced circulating IgM memory B cells and disappearance of intestinal IgA-secreting plasma cells. CVID patients with reduced circulating IgM memory B cells had a reduced frequency of gut IgA^+^ plasma cells and a disrupted film of SIgA on epithelial cells. Toll-like receptor 9 (TLR9) and transmembrane activator and calcium-modulator and cyclophilin ligand interactor (TACI) induced IgM memory B cell differentiation into IgA^+^ plasma cells *in vitro*. In the human gut, TACI-expressing IgM memory B cells were localized under the epithelial cell layer where the TACI ligand a proliferation inducing ligand (APRIL) was extremely abundant.

**Conclusions:** Circulating IgM memory B cell depletion was associated with a defect of intestinal IgA-secreting plasma cells in asplenia and CVID. The observation that IgM memory B cells have a distinctive role in mucosal protection suggests the existence of a functional gut-spleen axis.

## Introduction

B cells produce antibodies of different isotypes with defined effector functions. Secretory immunoglobulin A (SIgA) is used by B cells to protect mucosal sites (1). Whereas, serum antibodies act after pathogen invasion, SIgA is localized on the external surface of epithelial cells in direct contact with commensal and pathogenic microorganisms. In the gut, SIgA regulates the diversity of the microbiota (2, 3) and modulates immune activation by enteric commensals and food antigens (4). Moreover, SIgA, by controlling intestinal homeostasis, plays a role in the prevention of bacteria-driven inflammatory, autoimmune, and neoplastic B cell pathology (5). SIgA has a similar distribution and function in the respiratory tract (6) where it prevents bacterial colonization and carriage (7). IgA exists as a monomer in the serum and as a dimer in secretions. The Joining (J) chain covalently linking two monomers forms dimeric IgA. The polymeric immunoglobulin receptor (pIgR) expressed on the basolateral surface of mucosal epithelial cells binds to the J chain and transports dimeric IgA to the apical membrane. Here the external domain of the pIgR is cleaved by proteolysis thus releasing dimeric IgA bound to the fragment of pIgR called secretory component (SC) (8). In the mouse, the majority of SIgA lining the intestinal epithelium corresponds to natural antibodies, produced without intentional immunization in a thymus-independent manner by B-1a B cells. We have previously demonstrated that both B-1a B cells and SIgA are absent in the gut of asplenic mice (9). In humans, the population of B cells, known as innate IgM memory B cells, natural memory, natural effector, marginal zone B cells, is functionally similar to mouse B-1a B cells. Innate IgM memory B cells are generated from transitional B cells through Toll-like receptor (TLR) 9 stimulation *in vitro* (10–13) and can be found in patients with hyper IgM type 1 syndrome and in those with severe combined immune deficiency (14–16). While switched memory B cells are generated by previous immune responses in the germinal centers (GCs) independently from the presence of the spleen, IgM memory B cells may belong to a separate lineage (16, 17). They are found in the spleen (18) and in the peripheral blood, are generated through a T cell- and GC-independent mechanism (19), and respond to polysaccharides of encapsulated bacteria. IgM memory B cells are reduced after splenectomy (20). It has been shown that gut IgM^+^ and IgA^+^ plasma cells are clonally related to a large repertoire of IgM memory B cells disseminated throughout the intestine (21). In the intestine, IgA class switching is mediated by two different mechanisms, one dependent and one independent on T cells. T-cell dependent SIgA is generated by the adaptive immune response in the GCs of mesenteric lymph nodes and Peyer patches (22). IgA class switch can occur in a T cell-independent manner in the lamina propria (23, 24) and in the gut-associated lymphoid tissue (25, 26), as demonstrated in patients with CD40 ligand deficiency (23). In T cell-independent IgA class switch (27, 28), an important role is played by the interaction between the transmembrane activator and calcium-modulator and cyclophilin ligand interactor (TACI) and its ligand a proliferation inducing ligand (APRIL) (29). This phenomenon occurs in a MyD88/IRAK4-dependent manner (30). Here, we investigate the gut mucosa of two distinct clinical conditions only sharing the reduction of circulating IgM memory B cells, i.e., splenectomized patients and patients affected by CVID (31). We show that patients with low numbers of circulating IgM memory B cells have a reduced frequency of IgA^+^ plasma cells in the gut and a disrupted film of SIgA on epithelial cells. We also show that *in vitro* IgM memory B cells are the only B cell type able to respond to TLR9 and TACI cross-linking by differentiating into IgA^+^ plasma cells.

## Results

### Intestinal Secretory Immunoglobulin A Is Reduced After Splenectomy

**Figure 1:**
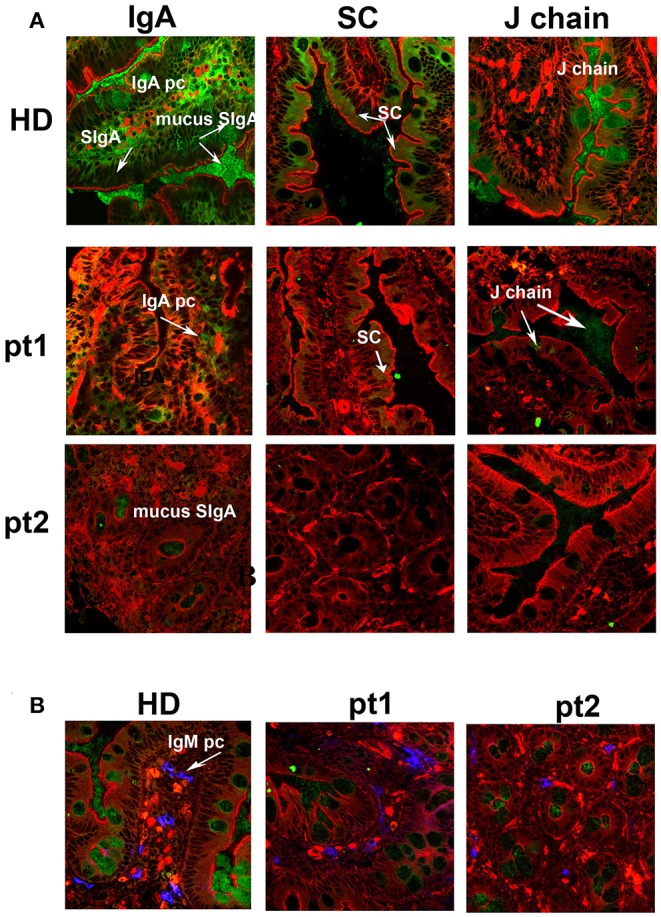
Secretory immunoglobulin A (SIgA) is reduced in splenectomized patients. **(A)** Cryostat sections of the duodenum were stained in red with phalloidin (a toxin that binds and stains filamentous actin) and in green with antibodies against either IgA (left panels), secretory component (SC, middle panels), or J-chain (right panels). Biopsies of one healthy donor (HD) and two representative splenectomized patients (pt.1 and pt.2) are shown. A reduction of IgA^+^ plasma cells, SIgA, SC, and J chain is observed after removal of the spleen. **(B)** IgM^+^ plasma cells are shown in blue along the axis of the villi in HD and asplenic patients. IgM is not significantly secreted.

We and others have previously shown that removal of the spleen causes the reduction of IgM memory B cells in the peripheral blood (12, 20, 32). In order to verify whether IgM memory B cells might have a role in the mucosal protection, we analyzed duodenal biopsies of seven patients who had been splenectomized because of traumatic rupture of the spleen and did not show any pre-existing immune, hematologic, or neoplastic comorbidities. They underwent upper endoscopy to investigate dyspepsia. All of them had serum Ig levels within the normal range (Supplementary Table 1). The number of CD27^+^ IgM and switched memory B cells was reduced in comparison to healthy donors (HD, *n* = 51). Absolute counts for CD27^+^ IgM^+^ B cells were 17 ± 11 cells/mm^3^ (normal value 55 ± 35 cells/mm^3^, *p* = 0.003), while absolute counts for CD27^+^ switched memory B cells were 29 ± 17 cells/mm^3^ (normal value 58 ± 37 cells/mm^3^, *p* = 0.6) (Supplementary Table 1). Cryostat sections stained with phalloidin, in order to visualize the tissue architecture, and with antibodies against IgA, were analyzed by confocal microscopy. In the HD cohort, IgA^+^ plasma cells appeared as bright and large green cells in the axis of the villi and beneath the epithelial cell layer in the crypts (Figure 1A, IgA panel). SIgA was transported through the epithelial cells to the luminal surface where it remained in the mucus. IgA transport can be tracked by staining the SC with a specific antibody. The pIgR fragment became visible toward the luminal side of the epithelial cells after the enzymatic cleavage that released the SC bound to IgA into the lumen while directing the rest of the pIgR to the recycling pathway (Figure 1A, SC panel). The J chain was only detected in the mucus because the epitope identified by the antibodies we used was not accessible either in plasma cell cytoplasm or when the J chain was bound to the intact pIgR (Figure 1A, J chain panel). Furthermore, HD IgM^+^ plasma cells were visualized as bright and large blue cells in the axis of the villi and beneath the epithelial layer in the crypts, while secretory IgM (SIgM) was not evident at the luminal side of the epithelial cells (Figure 1B, IgM panel). We counted plasma cells in the villous axis in well-oriented duodenal biopsies by considering seven villi per patient in three different slides. In splenectomized subjects, intestinal IgA^+^ plasma cells were reduced to half of normal (6.8 ± 4.0 vs. 15.0 ± 0.7 plasma cells/villus, *p* < 0.0001), and SIgA was not significantly transported through the epithelial cells. In some cases, patches of SIgA scattered in the lamina propria were observed (Figure 1A, IgA panel). Both SC and J chain were hardly visible (Figure 1A, SC and J chain panels). IgM was not significantly transported to the luminal side of the epithelial cells, and IgM^+^ plasma cells were slightly reduced in splenectomized patients (Figure 1B, IgM panel). Thus, removal of the spleen was associated not only with a reduction of circulating IgM memory B cells, but also with the disappearance of the SIgA film on epithelial cells.

### Intestinal Secretory Immunoglobulin A Are Reduced in Common Variable Immune Deficiency Patients With Circulating Immunoglobulin M Memory B Cell Depletion

We have previously shown that in CVID patients the frequency and numbers of circulating switched memory B cells are always reduced, but IgM memory B cells may be preserved (31). Patients with a severe defect of IgM memory B cells suffer from recurrent respiratory infections mostly caused by encapsulated bacteria, with a possible evolution in chronic lung diseases (33). In contrast, CVID patients with normal frequencies of IgM memory B cells have a lower incidence of infections. Moreover, serum IgA has a protective role against respiratory infections as demonstrated by the increased risk of pneumonia in CVID patients with IgA serum levels lower than 7 mg/dl (33). Here, we analyzed a cohort of 33 CVID patients and 51 HD. We stratified CVID patients according to the number of peripheral IgM memory B cells. Group 1 (22/33) had an absolute number of IgM memory B cells <20 cells/mm^3^ (6 ± 6 cells/mm^3^), whereas Group 2 (11/33) had a number of IgM memory B cells >20 cells/mm^3^ (105 ± 110 cells/mm^3^).

Switched memory B cells were reduced in both groups, with Group 1 showing significantly lower levels than Group 2 (2 ± 3 cells/mm^3^ vs. 17 ± 24 cells/mm^3^, *p* = 0.006). Serum IgM concentration was significantly lower in Group 1 (25.5 ± 68.3 mg/dl) as compared to Group 2 (30.8 ± 19.6 mg/dl, *p* = 0.009). Serum IgA levels were also reduced in Group 1 in comparison to Group 2 (5.4 ± 11.8 mg/dl vs. 23.1 ± 60.7 mg/dl), but the difference did not reach significance (*p* = 0.06). Serum IgG levels at diagnosis (208.4 ± 130.4 vs. 267.7 ± 118.4 mg/dl) were comparable (Supplementary Table 2).

The analysis of levels of specific anti-pneumococcus capsular polysaccharide (PCP) IgM and IgA before and after pneumococcal vaccination revealed no difference before immunization between Groups 1 and 2 for both specific anti-PCP IgM (3.6 ± 8.8 vs. 8.9 ± 9.7 UI/ml, *p* = 0.2) and IgA (2.1 ± 3.1 vs. 4.2 ± 4.3 UI/ml, *p* = 0.2). Post immunization anti-PCP IgM and IgA were reduced in both groups in comparison to HD (*p* < 0.0001). Group 1 showed significantly lower anti-PCP IgM (2.3 ± 3.7 UI/ml) than Group 2 (12.2 ± 12.6 UI/ml, *p* = 0.02). Group 1 post anti-PCP IgA was lower (1.4 ± 1.7 UI/ml) than Group 2 post anti-PCP IgA (92.2 ± 172.8 UI/ml), even if this difference was not statistically significant due to the high SD (Supplementary Table 2).

The clinical picture of the two groups was different, with Group 1 displaying a more severe phenotype. Recurrent pneumonia episodes and bronchiectasis were reported more frequently in Group 1 than in Group 2 (59 vs. 9%, *p* = 0.009 and 73 vs. 27%, *p* = 0.02, Supplementary Table 3). Gastrointestinal symptoms were also described more commonly in Group 1 than in Group 2 (63 vs. 18%, *p* = 0.02). In particular, recurrent episodes of diarrhea were reported in 59% of patients from Group 1 and in 18% from those in Group 2 (*p* = 0.03). Detailed gastrointestinal diagnosis and gut alteration have been summarized in Supplementary Table 3.

Patients from Group 1 had frequently more splenic alteration in comparison to those in Group 2 (77 vs. 36%, *p* = 0.05). Interestingly, subjects who had severe splenomegaly (six patients) or who had been splenectomized (four patients) were all classified as Group 1 (Supplementary Table 3). Patients with splenomegaly showed lower number of IgM memory B cells and switched memory B cells in comparison to those without spleen alteration as summarized in Supplementary Figure 1.

**Figure 2:**
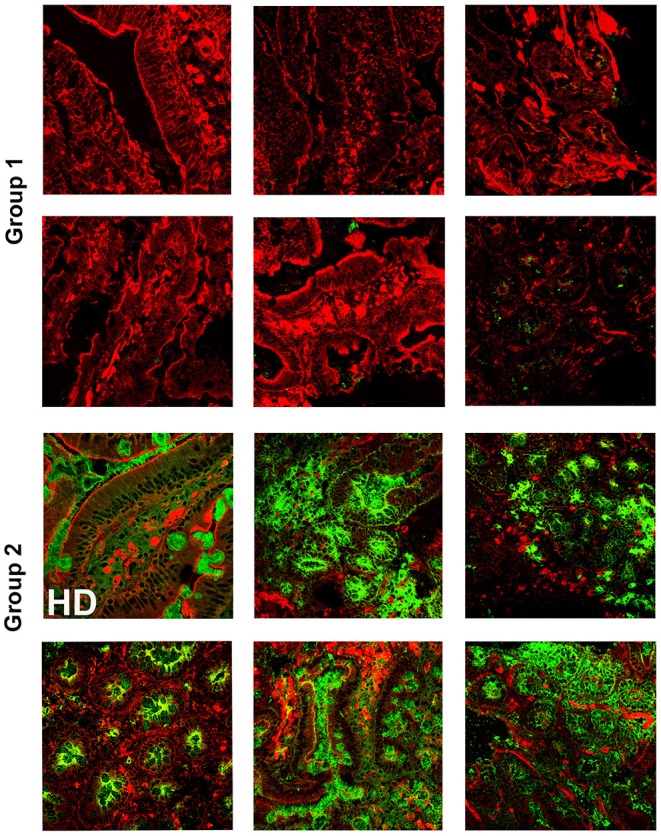
Lamina propria expression of secretory immunoglobulin A (SIgA) in the gut of CVID patients who have reduced (Group 1) or normal (Group 2) number of circulating immunoglobulin M (IgM) memory B cells. Cryostat duodenal sections were stained with phalloidin (red) and immunoglobulin A (IgA) (green) in six patients of Group 1 with reduced numbers of circulating IgM memory B cells **(upper panels)**, and a control subject (HD) together with five patients belonging to Group 2 with normal numbers of circulating IgM memory B cells **(lower panels)**.

**Figure 3:**
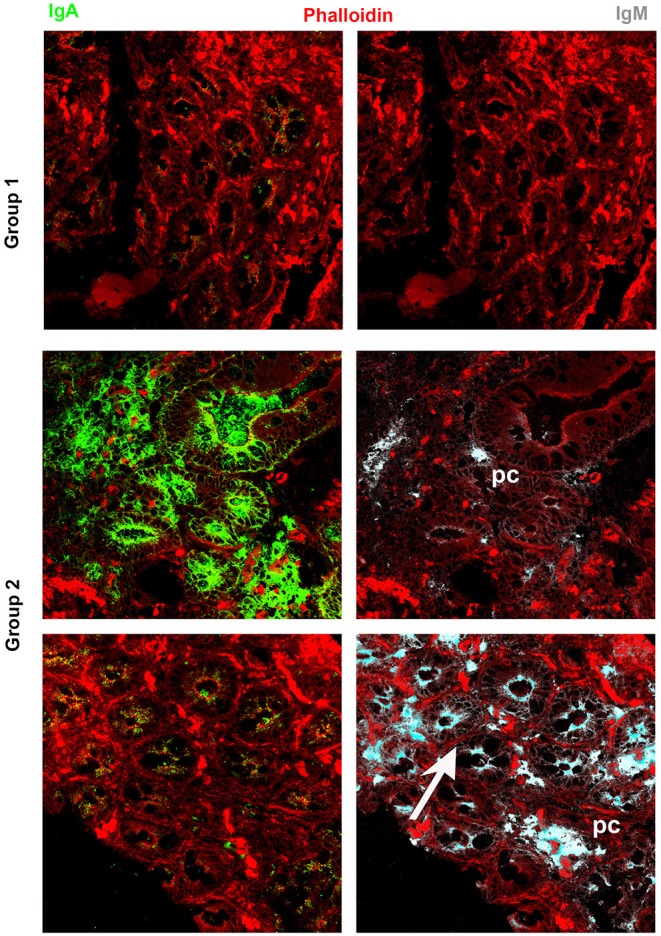
Immunoglobulin M (IgM) can substitute immunoglobulin A (IgA) in the gut of common variable immune deficiency (CVID) patients. Cryostat sections were stained with phalloidin (red), IgA (green), and IgM (gray). IgA **(left panels)** and IgM **(right panels)** staining in the same sections are shown separately. In CVID patients belonging to Group 1, neither IgA nor IgM is expressed. In most CVID patients belonging to Group 2, IgA is the major isotype expressed and transported (pt.22, as representative example). IgM substitutes IgA in one case (pt.30, **lower panel**) and is transported through epithelial cells.

It has been widely recognized that gastrointestinal infections and chronic intestinal inflammation are common in CVID (34–36) and are associated with high morbidity (37). Moreover, CVID patients have an increased risk of gastric cancer (38), and therefore surveillance gastroscopy with collection of biopsies is regularly performed in our center according to the Italian guidelines (www.ipinet.org). We excluded from the study CVID patients with a histological report of an abnormal tissue architecture (i.e., villous atrophy). We analyzed the presence of IgA^+^ plasma cells, IgM^+^ plasma cells, and the distribution of SIgA and sIgM in the duodenal samples of the two CVID groups (Supplementary Table 2). We counted plasma cells in the villous axis in well-oriented duodenal biopsies by considering seven villi per patient in three different slides. Only one patient of Group 1 had IgA^+^ plasma cells in the gut, whereas IgA^+^ plasma cells were visible in eight patients (73%) of Group 2 (Figure 2). Only one patient of Group 1 (1/22, 4.5%) had IgM^+^ plasma cells in the intestine, whereas IgM^+^ plasma cells were visible in six patients (6/11, 54.5%) of Group 2 (Figure 3, IgM, middle panel). No patient of Group 1 had SIgA in the intestine, whereas SIgA were found in the same patients of Group 2 who had IgA^+^ plasma cells (Figure 2). In only two patients of Group 2, sIgM were detectable in the microfilm of mucus on epithelial cells (Figure 3, IgM, lower panel). In summary, we found that a low number of circulating IgM memory B cells were associated with a severe defect of mucosal SIgA.

### Toll-Like Receptor 9 and Transmembrane Activator and Calcium-Modulator and Cyclophilin Ligand Interactor Cooperatively Induce Class Switching of Immunoglobulin M Memory B Cells to Immunoglobulin A

**Figure 4:**
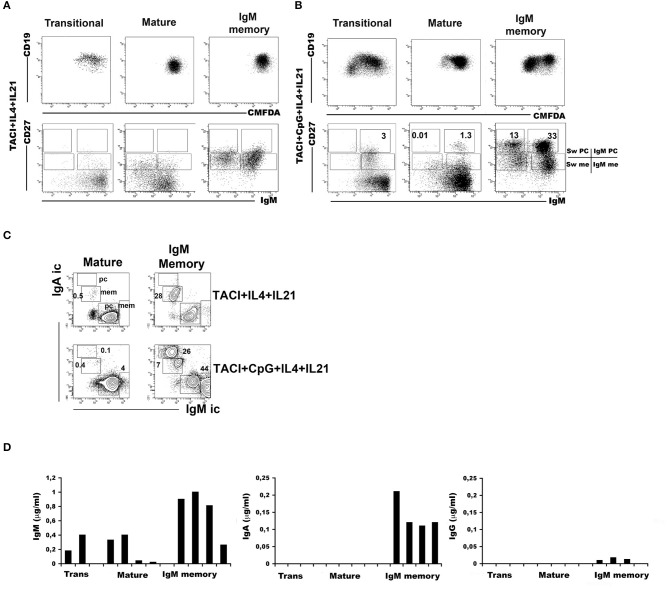
Immunoglobulin M (IgM) memory B cells differentiate into IgA^+^ plasma cells in response to transmembrane activator and calcium-modulator and cyclophilin ligand interactor (TACI) and toll-like receptor 9 (TLR9) co-stimulation. Flow-cytometry analysis after 7 days in culture with anti-TACI plus IL-4 and IL-21. The loss of intracellular CMFDA tracks cell division. **(A)** No proliferation is observed in mature-naive and IgM memory B cells, whereas a modest expansion of transitional B cells is observed (upper panels). The IgM/CD27 staining (lower panels) shows the loss of surface IgM in 17.0% of mature-naïve and 43.6% of IgM memory B cells (bottom panels). **(B)** The addition of CpG induces proliferation as shown by the loss of CMFDA fluorescence, mainly in transitional and IgM memory B cells (top panels). Differentiation into IgM^+^ CD27^+^ plasma cells is observed in all B cell types. Switched plasma cells are only generated from IgM memory B cells. **(C)** After 7 days with anti-TACI, a small fraction of mature-naïve B cells had switched to IgA. The addition of CpG further increased survival. Upon TACI cross-linking, almost half of the IgM memory B cells switched to IgA, but when CpG was added to the culture, IgM memory B cells differentiated into IgA^+^ plasma cells. **(D)** Graphs indicate the concentration of IgM and IgA (ng/ml) detected by ELISA in culture supernatants obtained from B cells stimulated with anti-TACI, CpG, IL-4, and IL-21. IgM can be detected in the supernatants of all B cell types, but only stimulated IgM memory B cells secreted IgA. IgG production was never detectable.

Transitional, mature-naïve and memory B cells all express TACI on the cell-surface. The highest expression is observed in IgM memory B cells and the lowest in switched memory B cells from peripheral blood (Supplementary Figure 2A). Stimulation of TLR9 through its ligand CpG upregulates TACI expression on naïve B cells and IgM memory B cells. TACI expression remains low in switched memory B cells and undetectable on newly generated plasma cells (Supplementary Figure 2B). We asked whether all B cell types are able to switch to IgA upon TACI engagement and investigated the effects of co-stimulation with the TLR9 ligand CpG. The response of B cell populations to CpG had been previously investigated (10). IgM memory B cells proliferate and differentiate into plasma cells secreting high amount of IgM and barely detectable IgG, but never IgA. Mature-naïve B cells survive without differentiation. Transitional B cells proliferate and differentiate into IgM memory B cells and IgM^+^ plasma cells (10, 13). In most normal adults, the population of switched memory B cells is composed of 2/3 by IgG expressing memory B cells with the remaining 1/3 expressing IgA. All switched memory B cells expand upon stimulation with CpG and differentiate into IgG^+^ and IgA^+^ plasma cells (39). Cooperation between TLR9 and TACI is possible because the two receptors share the same adaptor protein, MyD88 (30). Moreover, APRIL, the ligand for TACI, is expressed in the gut (22, 24), where the microbiota provides TLR ligands in abundance. We studied the effects of TACI or TACI in combination with CpG in mature-naïve (CD19^+^ CD24^+^ CD27^−^) or IgM memory B cells (CD19^+^ CD24^+^ CD27^+^ IgG^−^ IgA^−^) sorted from the peripheral blood of adult donors. In all cultures, we included interleukin IL-4 and IL-21, a combination of cytokines that increases survival of mature-naïve B cells and plasma cell formation without changing the quality of the response. Plasma cells can be distinguished from memory B cells based on their higher CD27 expression and can express either IgM (IgM plasma cells) or other isotypes (switched plasma cells). After 7-day culture with anti-TACI, neither mature-naïve nor IgM memory B cells generated plasma cells (Figure 4A). A small fraction of mature-naïve B cells and a large one of IgM memory B cells switched to IgA. This is suggested by the loss of surface IgM (Figure 4A) and demonstrated by the expression of intracellular IgA in 0.5% of the mature and in 28% of the IgM memory populations (Figure 4C, TACI+IL-4+IL-21). When CpG was added to the cultures, IgM memory B cells differentiated into IgM^+^ and IgM^−^ plasma cells (Figure 4B, TACI+CpG+IL-4+IL-21) containing high amounts of intracellular IgM or IgA. Only few IgM^+^ (4%) and even less IgA^+^ (0.1%) plasma cells were obtained from mature B cells (Figure 4C) in the same conditions. Transitional, mature-naïve and IgM memory B cells stimulated with CpG, anti-TACI and IL-4/IL-21 all produced IgM. IgA was detected only in the cultures containing IgM memory B cells (Figure 4D). In summary, we concluded that only IgM memory B cells differentiate into IgA^+^ plasma cells in response to TACI and CpG.

### Expression of a Proliferation Inducing Ligand, CD27, and Immunoglobulin M in the Adult Gut

**Figure 5:**
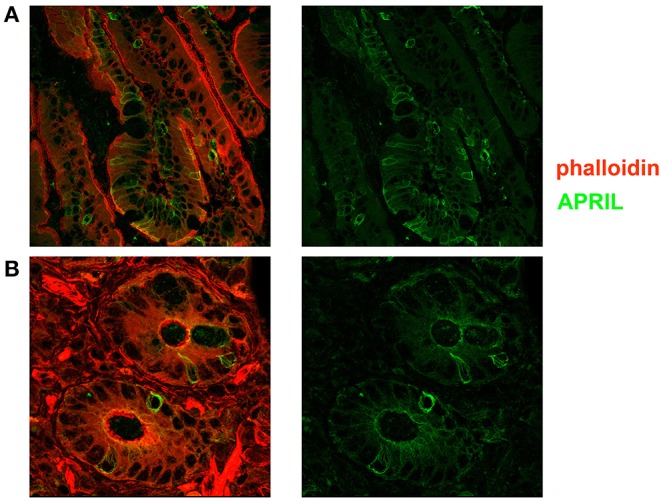
Expression of a proliferation inducing ligand (APRIL) in the adult gut. Cryostat sections from adult intestine were stained for actin (phalloidin, red) and APRIL (green). **(A)** We show the overlay of phalloidin and APRIL staining (left) and APRIL alone (right). APRIL can be detected in the epithelium, but is not equally expressed by each epithelial cell, probably reflecting the topographic distribution of inductive signals (magnification ×60). **(B)** Details of cryptae analyzed with ×60 objective amplification are shown. We observe a gradient of APRIL expression starting at the luminal side and progressing toward the basal side of the epithelial cell.

**Figure 6:**
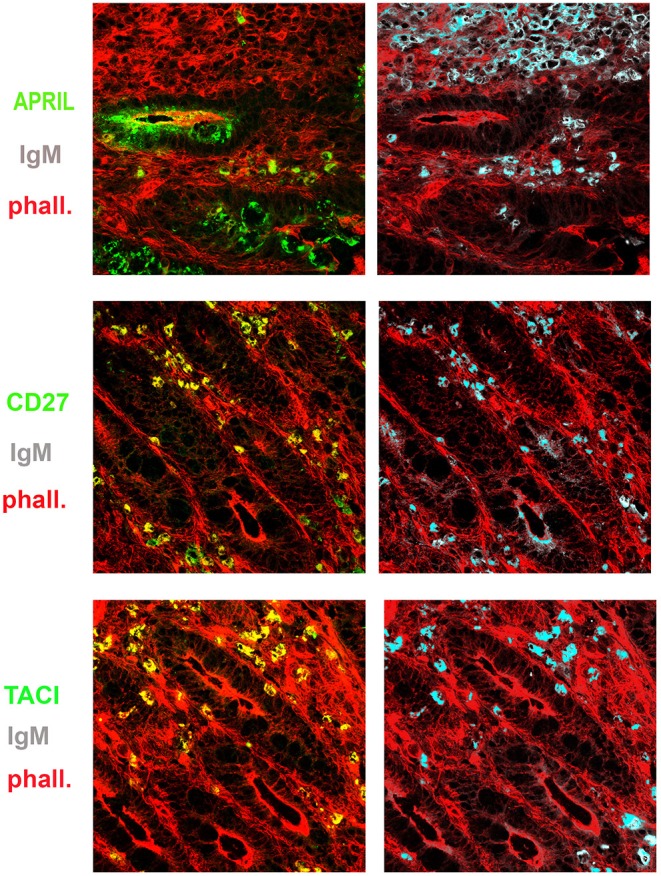
Expression of a proliferation inducing ligand (APRIL), transmembrane activator and calcium-modulator and cyclophilin ligand interactor (TACI), CD27, and immunoglobulin M (IgM) in the gut. Cryostat sections from the intestine were stained for actin (phalloidin, red) and APRIL (green) **(upper panel)**. APRIL is expressed by epithelial cells of the crypts. Intestinal sample is stained for CD27 (green) and IgM (gray) **(middle panel)**. From the same field (×20 magnification) we show in separate panels the overlay of phalloidin, TACI, and IgM. CD27^+^ (left) IgM^+^ (right) B cells were distributed among the crypts (middle panels). IgM^+^ B cells express TACI **(lower panel)**.

Recently, Magri et al. (21) demonstrated that IgM^+^ plasma cells in the gut are clonally related to IgM memory B cells disseminated throughout the intestine. They also showed that IgM memory B cells switch to IgA in response to T-independent and dependent signals *in vitro*. We asked the question whether IgM memory B cells expressing TACI could be found in the intestine in the vicinity of the TACI-ligand APRIL. We showed that in the adult gut, APRIL can be detected in the epithelium, and it is expressed by crypt epithelial cells but is not equally expressed by each epithelial cell, probably reflecting the topographic distribution of inductive signals. We observed a gradient of APRIL expression starting at the luminal side and progressing toward the basal side of the epithelial cell (Figure 5). B cells were localized under the epithelial cell layer where APRIL was extremely abundant. In addition, IgM^+^ B cells expressed TACI (Figure 6). An indication that B cells follow a migratory pathway connecting spleen and intestine has been suggested before based on the metastatic behavior of marginal zone lymphomas, as also shown in one of our CVID patients who developed a marginal zone B-cell lymphoma of the spleen, a tumor thought to originate from memory B cells expressing CD27 and IgM. We observed large numbers of CD27^+^ IgM^+^ B cells in the intestinal biopsy of this CVID patient of Group 1. Differently from the organized distribution localized around the cryptae and along the axis of the villi observed in HD, in the intestinal biopsies taken before splenectomy, clusters of CD27^+^ IgM^+^ cells were observed throughout the tissue, infiltrating and disrupting the structure (Supplementary Figure 3).

## Discussion

SIgA is a pillar of the mucosal barrier against bacterial dissemination, it prevents adhesion and penetration of antigens, and neutralizes biologically active substances and viruses forming complexes in antigen- and non-antigen specific manner by interacting with IgA Fc receptors (40, 41). After bacterial sampling by dendritic cells or invasion, pathogens transported to the local lymph nodes induce the formation of GCs. Highly specific B cells then migrate back to the submucosa into the Peyer's patches and here differentiate into IgA-producing plasma cells. In the lamina propria, B cells may also switch into IgA in a T cell-independent way (21). The T cell-independent generation of SIgA by B cells may represent a mechanism to generate IgA expressing a wide repertoire of Ig genes, useful to face the thousands of different bacterial species of the microbiota (23, 24, 42), and to control host-microbiota mutualism, reducing the risk of bacterial translocation and immune activation (43–45). IgM memory B cells have been shown to home to the gut and to locally switch to IgA (21, 46). Since IgM memory B cells are reduced in patients who had been splenectomized or are affected by CVID (20, 31), we asked the question of whether the reduction of this B cell subset might influence the production of SIgA in the gut. We analyzed patients with an intact and functional immune system that had been splenectomized because of injury and patients affected by CVID, a primary antibody deficiency. In splenectomized patients, the T cell-independent responses to polysaccharide antigens were impaired (47) but the adaptive immune response in the lymph nodes remained functional and serum Ig levels were normal. We showed that splenectomy does not affect the number of peripheral naive B, but causes a reduction of IgM memory B cells, as previously shown (12, 20, 48). The reduction of IgM memory B cells in the peripheral blood was associated with the reduction of IgA^+^ plasma cells in the gut. In particular, our patients did not have pre-existing diseases, and splenectomy was due to traumatic rupture of the spleen. If SIgA in the intestine was generated by local immune responses initiated either by transitional or naïve B cells, a reconstruction of the SIgA layer should be observed after splenectomy. This did not happen even years (few months to 15 years) after splenectomy. Thus, the responses occurring in the organized lymphoid tissue of the gut and in the lymph nodes throughout the body were not sufficient to fully re-construct the continuous film of SIgA in the gut. Of note, these findings may provide a pathophysiological explanation for the gut microbiota alterations over time after splenectomy and the higher susceptibility observed in splenectomized patients to infections sustained by enteric bacteria, including *Enterococci, Escherichia coli* (*E. coli*), and other enterobacteria (49, 50).

Patients with CVID had reduced serum Ig levels and were unable to mount an effective antibody response (37). Antibodies of IgG isotype are regularly administered in order to transfer the antigenic experience of donors. However, despite IgG replacement, the subset of CVID patients having reduced frequency of IgM memory B cells, reduced number of switched memory B cells and a very low IgA level had a severe infection risk (33). Moreover, CVID patients suffered from infectious and inflammatory disorders (34, 36), all conditions affecting patients' morbidity and quality of life (51). In CVID patients, the shift in the gut microbial composition and the reduced diversity of the gut microbiota promoted immune activation and inflammation, without obvious associations with antibiotic use (52). Spleen involvement was frequent in CVID. Splenomegaly had been reported in one out of four patients enrolled in the database of the European Society for Immunodeficiencies (ESID), but neither its causes nor its consequences were well-understood. Splenomegaly is associated with autoimmunity, granulomas but also with liver disease and portal hypertension (53). Here, we observed that patients with splenomegaly had reduced IgM memory and switched memory B cells, confirming data from other cohorts (54, 55).

The majority of CVID patients with a normal number of IgM memory B cells had a visible layer of SIgA, while all CVID patients with low numbers of IgM memory B cells lacked SIgA. The observation that at least one of our patients could substitute SIgA and IgA^+^ plasma cells with sIgM and IgM^+^ plasma cells may explain why selective IgA deficiency can remain asymptomatic. T cell-independent IgA class switch is promoted by the interaction between TACI and its ligand APRIL through up-regulation of AID expression (27–29). We show that IgM memory B cells are able to develop into IgA^+^ plasma cells *in vitro* upon stimulation with TLR9 and TACI. TACI, differently from other members of the TNF receptor family, is able to cooperate with TLR9. Protection of mucosal sites from colonizing bacteria has been a prerequisite for life throughout evolution. The innate immune system evolved first. B and T cells appeared in fish. Fish have no bone marrow, lymph nodes or GCs, but they have the spleen and in the gut they produce an antibody called IgT, dimeric as IgA and transported to the intestinal lumen by the pIgR (56, 57). Mice have SIgA generated by B-1a B cells, independently of T cells. It is attractive to hypothesize that a primitive defense system still exists in man, and IgM memory B cells belong to it. Further studies are necessary to identify the cellular and molecular mechanisms used by colonizing bacteria to trigger the development of IgM memory B cells in the spleen, their migration to the gut, and the organization of local immunity. Recently, it has been demonstrated that IgM^+^ plasma cells in the gut are clonally related to IgM memory B cells disseminated throughout the intestine (21), and that IgM memory B cells switch to IgA in response to T-independent and dependent signals *in vitro*. We confirm that TACI-expressing IgM memory B cells are localized under the epithelial cell layer. Epithelial cells, in turn, express the TACI ligand APRIL.

In conclusion, our results suggest that IgM memory B cells may play a distinctive role in mucosal protection by migrating to the gut where they switch to IgA. In the absence of IgM memory B cells, naïve or transitional B cells, which are both present and functional in our group of splenectomized patients, are not able to regenerate the SIgA film and to replenish the IgA plasma cells in the gut. New tools should be developed in order to substitute the function of SIgA in asplenia and in primary antibody deficiencies, and further studies are necessary to confirm the existence of a functional gut-spleen axis.

## Materials and Methods

### Patients

Seven patients, who had been splenectomized for trauma, without pre-existing comorbidities were enrolled into the study as they had undergone an upper endoscopy for investigating a dyspepsia. Patients affected by CVID (*n* = 33) were diagnosed according to the ESID/PAGID criteria (26). Patients with CVID have an increased risk of gastric cancer, and for this reason the Italian guidelines include annual endoscopy with biopsy collection. All patients were on intravenous or subcutaneous immunoglobulin substitution therapy with trough IgG serum levels above 500 mg/dl according to the national guidelines. CVID participants could also be treated with additional drugs following consolidated clinical practice and guidelines. However, no patient was receiving either steroid treatment or immunosuppressive therapy. None of the patients included in this study had TACI mutations, and all CVID patients with architectural abnormalities of the duodenal mucosa at the study time were excluded from the study. Fifty-one HD were enrolled as controls for blood values and 15 HD for intestinal biopsies. For 19 patients and 20 HD we assessed by ELISA test the levels of specific anti PCP-IgM and anti PCP-IgA before and after pneumococcal vaccination by one dose of a 23-valent polysaccharide vaccine (Pneumovax®) as described by Cavaliere et al. (58). The choice to measure IgM and IgA anti-PCP instead of IgG, was due to the necessity to overcome the impossibility of studying vaccine responses in subjects on IgG replacement therapy. Protocols were conformed to the ethical guidelines of the 1975 Declaration of Helsinki as reflected in *a priori* approval by the institution's human research committee.

### Flow Cytometry Analysis

Peripheral blood mononuclear cells were isolated and stained as previously described (12). Briefly, mature-naïve B cells are (CD24^+^CD27^−^IgM^+^IgD^bright^). Two populations of memory B cells can be identified: IgM memory B cells (CD24^+^CD27^+^IgM^+^IgD^dull^) and switched memory B cells (CD24^+^CD27^+^IgM^−^IgD^−^).

### Cell Sorting

Peripheral blood mononuclear cells were isolated from heparinized peripheral blood by Ficoll-Paque^TM^ Plus (Amersham Biosciences, Little Chalfont, UK) density-gradient centrifugation and were stained with the following antibodies: clone ML5 (anti-CD24), clone M-T271 (anti-CD27), clone G18-145 (anti-IgG), and streptavidin-APC-Cy7 were obtained from BD Biosciences (San Diego, CA, USA) and clone B35C6B4 (anti-IgA_1_) and clone A964D2 (anti-IgA_2_) from Southern Biotechnologies. After staining the lymphocytes were sorted as mature-naive B cells (CD24^+^CD27^−^) and IgM memory B cells (CD24^+^CD27^+^IgG^−^IgA^1−2−^) using a FACSvantage SE (Becton and Dickinson, Sunnyvale, California, USA). A negative gating strategy was used to sort IgM memory B cells in order to avoid B-cell activation through the BCR. Dead cells were excluded from analysis by side/forward scatter gating and cell purity was >98%. Cord blood mononuclear cells were stained for CD24 and CD38 and sorted for transitional B cells (CD24^bright^CD38^bright^) as described above.

### Cell Culture

Transitional, mature naive, and IgM memory sorted B cells were labeled with 5-Chloromethyl fluorescein diacetate at the final concentration of 0.1 mg/ml (CellTracker CMFDA, Molecular Probes, Eugene, OR) and cultured in 96 well plates (Becton Dickinson) with RPMI 1640 (Gibco BRL), 10% heat inactivated fetal bovine serum (FBS, Hyclone Laboratories Logan UT), 2% L-glutamine (Gibco BRL), 5 × 10–5 M 2-βmercaptoethanol (Sigma, St. Louis, USA) and 20 mg/ml gentamycin (Gibco BRL), supplemented with either 2.5 μg/ml CpG-ODN17 (Hycult Biotechnology, Uden, The Netherlands), 20 ng/ml IL-21 (Peprotech, UK), 20 ng/ml IL-4 (Peprotech, UK), 1 μg/ml anti-human CD267 (anti-TACI) (eBioscience, San Diego, CA, USA), and beads coated with anti-mouse IgG (Dynal Life Technologies Europe) at the proportion of one bead per 50 cells. Cell proliferation and phenotypic analysis were performed on day 7 by flow-cytometry using a FACSCalibur Flow Cytometer (BD Biosciences). Secreted Igs were detected in the supernatants at day 7 by ELISA.

### Enzyme-Linked Immunosorbent Assay

Briefly, 96-well plates were coated overnight with purified goat anti-human IgA, IgG, or IgM antibodies (Jackson Immuno Research Laboratories, Pennsylvania, USA). After washing and blocking, plates were incubated for 1 h with the supernatants of the cultured cells. After washing, plates were incubated for 1 h with peroxidase-conjugated fragment of goat antihuman IgA, IgG or IgM antibodies (Jackson Immuno Research Laboratories), and the assay was developed with o-phenylendiamine (Sigma-Aldrich). Optical density was measured on a microtiter plate reader at 450 nm and Ig concentrations were calculated by interpolation with the standard curve.

### Confocal Microscopy

Intestinal tissues were collected and immediately frozen in liquid nitrogen, and kept at −80°C until the time of the study. Multiple 5 μm cryostat sections obtained from frozen samples, included in cryostat embedding medium (Bio-Optica, Milan, Italy) were fixed in cold acetone, washed with PBS (Sigma, St. Louis, MO Sigma) and incubated for 45 min with Phalloidin-TRITC (70 mM, Sigma). Reagents used in the different stainings were FITC-anti-human- IgA, FITC-anti-human secretory component (Nordimmune, Tilburg, the Netherlands), FITC-antihuman J-chain (Mc19-9) (Serotec), anti-human CD256 (anti-APRIL, clone T3-6) (BioLegend, San Diego, CA, USA), anti-human CD267 (anti-TACI, clone 11H3) (eBioscience), TRITC-goat antihuman IgM, μ chain specific (Jackson Immunoresearch), and FITC-anti-human CD27 (clone MT271) obtained from BD Biosciences. Intestine sections were analyzed in a confocal microscope (Olympus FV1000), and images were acquired at ×20 and ×60 objective amplification. Three slides per patient were analyzed at the confocal microscope by four independent experts.

### Statistical Analysis

For comparison of clinical and biomarker changes between groups, the Student *T*-test and Mann-Whitney test were used for parametric or non-parametric datasets. Data were analyzed in the StatView statistical MacIntosh program (StatView Software, San Diego, CA). A level of *p* < 0.05 was considered statistically significant.

## Data Availability Statement

All datasets generated for this study are included in the article/Supplementary Material.

## Ethics Statement

The studies involving human participants were reviewed and approved by University of Rome, La Sapienza. The patients/participants provided their written informed consent to participate in this study.

## Author Contributions

RC, IQ, AD, MR and GC designed and coordinated the study, interpreted data, and wrote the manuscript. SC, EP, CM, OG, AA, EG and PB followed-up patients over time, locally collected data, made experiments, did statistical analysis, and reviewed the paper for final approval. All authors significantly participated in the drafting of the manuscript, critical revision of the manuscript for important intellectual content, and provided approval of the final submitted version.

### Conflict of Interest

The authors declare that the research was conducted in the absence of any commercial or financial relationships that could be construed as a potential conflict of interest.

## Abbreviations

AID: activation-induced cytidine deaminase
APRIL: a proliferation inducing ligand
CVID: common variable immune deficiency
HD: healthy donors
IL: interleukin
J: Joining
pIgR: polymeric immunoglobulin receptor
SIgA: secretory immunoglobulin A
SC: secretory component
TACI: transmembrane activator and calcium-modulator and cyclophilin ligand interactor
TLR: Toll-like receptor.
