# Supplementary material for: Lack of Gut Secretory Immunoglobulin A in Memory B-Cell Dysfunction-Associated Disorders: A Possible Gut-Spleen Axis
Source: Front Immunol. 2020 Jan 8;10:2937. doi: 10.3389/fimmu.2019.02937 (PMC6960143; doi:10.3389/fimmu.2019.02937)
Supplement: Supplementary file 1 [file Data_Sheet_1.docx]

**Supplementary Figure 1.** Bars indicate the number of (A) IgM memory B cells and (B) switched memory B cells (cell/mm^3^) in patients without spleen alteration, with splenomegaly and in those that were splenectomized. Statistical significances were calculated using unpaired, two-tailed Student’s *t*-tests: *p≤0.05

**Supplementary Figure 2**. Expression of TACI in B cell populations cultured in the absence or presence of CpG analyzed by flow-cytometry. (A) TACI is undetectable in switched memory and PCs. The geometric mean of fluorescence intensity and the standard deviations are indicated for each B cell subset, values were calculated from the TACI mean florescence intensity measured in four different donors analyzed in separate experiments. (B) TACI increases upon exposure to CpG in IgM memory and mature-naive B cells (B).

**Supplementary Figure 3.** Intestinal specimen from a group 1 CVID patient diagnosed with a marginal zone lymphoma stained for CD27 (green) and phalloidin (red). Monoclonal tumor cells staining for IgM (green) infiltrate the tissue disrupting the structure.

**Supplementary Table 1.** Demographic, clinical and immunological information on splenectomized patients. In all individuals the reason for splenectomy was traumatic rupture.

| **Patient ID** | **Age** | **Time from splenectomy (yrs)** | **IgG***  **mg/dl** | **IgM**  **mg/dl** | **IgA**  **mg/dl** | **B cells**  **(mm/3)** | **IgM memory**  **B cells**  **(mm/3)** | **Switched memory B cells**  **(mm/3)** | **SIgA*** | **IgA PC** | **SIgM*** | **IgM PC** |
| --- | --- | --- | --- | --- | --- | --- | --- | --- | --- | --- | --- | --- |
|  |  |  |  |  |  |  |  |  |  |  |  |  |
| 1 | 25 | 2 | 810 | 80 | 126 | 200 | 26 | 42 | + | - | - | + |
| 2 | 34 | 4 | 1895 | 38 | 129 | 220 | 6 | 19 | +/- | - | +/- | + |
| 3 | 32 | 1 | 648 | 22 | 125 | 360 | 36 | 61 | - | - | - | + |
| 4 | 27 | 0.25 | 423 | 88 | 122 | 172 | 15 | 22 | + | - | +/- | + |
| 5 | 69 | 2 | 2177 | 66 | 180 | 260 | 13 | 31 | + | - | +/- | - |
| 6 | 39 | 0.25 | 1944 | 96 | 126 | 300 | 15 | 17 | + | - | - | + |
| 7 | 54 | 15 | 419 | 65 | 110 | 228 | 6 | 14 | + | - | - | + |
|  |  |  |  |  |  |  |  |  |  |  |  |  |
| **Mean** |  |  | 1188 | 65 | 131.1 | 248.6 | 16.7 | 29.4 |  |  |  |  |
| **SD** |  |  | 781 | 26.7 | 22.4 | 64.1 | 10.8 | 16.9 |  |  |  |  |
| **HD Mean** |  |  | §600-1600 | §40-250 | §70-315 | 348 | 55 | 58 | +++ | + | +++ | ++ |
| **HD SD** |  |  |  |  |  | 155 | 35 | 37 |  |  |  |  |

*Arbitrary value established taking as reference for the minimum (-) an IgA deficient individual and for the maximum (+++) duodenal sections of healthy donors stained for IgA and IgM.

§ range

**Supplementary Table 2**. Demographic, clinical and immunological data on CVID enrolled in the study.

|  | **ID** | **Age** | **IgG at diagnosis** | **IgG TL** | **IgM** | **IgA** | **B cells** | **IgM memory B cells** | **Switched memory B cells** | **SIgA** | **IgA PC** | **SIgM** | **IgM PC** | **pre IgM anti-PC23** | **post IgM anti-PC23** | **pre IgA anti-PC23** | **post IgA anti-PC23** | **Pneumonia** | **Bronchiectasis** |
| --- | --- | --- | --- | --- | --- | --- | --- | --- | --- | --- | --- | --- | --- | --- | --- | --- | --- | --- | --- |
|  |  |  | **(mg/dl)** | **(mg/dl)** | **(mg/dl)** | **(mg/dl)** | **(mm/3)** | **(mm/3)** | **(mm/3)** |  |  |  |  | **(U/ml)** | **(U/ml)** | **(U/ml)** | **(U/ml)** |  |  |
| **GROUP 1** | **1** | 66 | 117 | 621 | 32 | 0 | 158 | 16 | 3 | - | - | - | - | 3.7 | 9.0 | 2.7 | 3.3 | Yes | Yes |
|  | **2** | 53 | 88 | 529 | 321 | 0 | 44 | 2 | 1 | - | - | - | - | nd | nd | nd | nd | Yes | Yes |
|  | **3** | 39 | 194 | 562 | 0 | 0 | 20 | 2 | 0 | - | - | - | - | 0.4 | 1.0 | 0.0 | 0.0 | Yes | Yes |
|  | **4** | 45 | 190 | 940 | 0 | 0 | 113 | 3 | 0 | - | - | - | - | 3.7 | 3.7 | 0.0 | 0.0 | Yes | No |
|  | **5** | 45 | 490 | 818 | 37 | 0 | 48 | 1 | 0.5 | - | - | - | - | 31.3 | 10.6 | 7.6 | 3.7 | Yes | No |
|  | **6** | 22 | 144 | 587 | 0 | 0 | 42 | 2 | 0 | - | - | - | - | 0.3 | 0.0 | 0.0 | 3.3 | No | Yes |
|  | **9** | 25 | 428 | 1020 | 60 | 46 | 529 | 5 | 16 | - | - | - | - | nd | nd | nd | nd | No | Yes |
|  | **10** | 24 | 433 | 549 | 30 | 0 | 174 | 9 | 2 | - | - | - | - | 3.2 | 2.7 | 8.8 | 2.7 | No | No |
|  | **13** | 58 | 220 | 850 | 5 | 10 | 19 | 2 | 1 | - | - | - | - | nd | nd | nd | nd | No | Yes |
|  | **14** | 54 | 150 | 740 | 0 | 0 | 844 | 8 | 0 | - | - | - | - | 0.3 | 0.3 | 0.0 | 0.0 | Yes | Yes |
|  | **17** | 36 | 396 | 522 | 42 | 28 | 14 | 2 | 1 | - | - | - | - | nd | nd | nd | nd | No | Yes |
|  | **18** | 43 | 120 | 583 | 0 | 0 | 7 | 0 | 0 | - | - | - | - | 0.0 | 0.0 | 0.0 | 0.0 | Yes | No |
|  | **20** | 67 | 220 | 664 | 24 | 6 | 60 | 19 | 2 | - | - | - | - | 0.3 | 0.3 | 0.0 | 0.0 | Yes | Yes |
|  | **21** | 21 | 192 | 718 | 0 | 0 | 349 | 7 | 0 | - | - | - | - | 0.4 | 0.3 | 3.9 | 3.7 | No | Yes |
|  | **23** | 47 | 53 | 720 | 0 | 0 | 32 | 4 | 1 | - | - | - | - | nd | nd | nd | nd | Yes | Yes |
|  | **24** | 61 | 310 | 860 | 0 | 0 | 109 | 1 | 2 | - | - | - | - | 0.0 | 0.0 | 0.0 | 0.0 | Yes | Yes |
|  | **25** | 67 | 182 | 620 | 5 | 22 | 49 | 17 | 3 | - | - | - | - | nd | nd | nd | nd | No | Yes |
|  | **26** | 58 | 269 | 799 | 0 | 0 | 195 | 14 | 0 | - | - | - | - | nd | nd | nd | nd | Yes | Yes |
|  | **28** | 37 | 55 | 787 | 0 | 0 | 27 | 0.3 | 0.3 | - | - | - | - | nd | nd | nd | nd | No | No |
|  | **29** | 70 | 129 | 706 | 0 | 0 | 212 | 4 | 2 | - | - | - | - | nd | nd | nd | nd | Yes | Yes |
|  | **31** | 35 | 185 | 700 | 4 | 6 | 32 | 1 | 1 | - | - | - | - | 0.0 | 0.0 | 2.8 | 0.0 | No | Yes |
|  | **15** | 23 | 20 | 595 | 0 | 0 | 51 | 1 | 2 | - | ++ | - | + | nd | nd | nd | nd | Yes | No |
|  |  |  |  |  |  |  |  |  |  |  |  |  |  |  |  |  |  |  |  |
|  | **Mean** | 45.3 | 208.4 | 704.1 | 25.5 | 5.4 | 142.2 | 5.7 | 1.9 |  |  |  |  | 3.6 | 2.3 | 2.2 | 1.4 |  |  |
|  | **SD** | 16.2 | 130.4 | 137.2 | 68.3 | 11.8 | 201.2 | 5.9 | 3.5 |  |  |  |  | 8.8 | 3.7 | 3.2 | 1.7 |  |  |
|  | **n.(%)** |  |  |  |  |  |  |  |  | 0 | 1 (4%) | 0 | 1 (4%) |  |  |  |  | 13 (59%) | 16 (73%) |

|  | **ID** | **Age** | **IgG at diagnosis** | **IgG TL** | **IgM** | **IgA** | **B cells** | **IgM memory B cells** | **Switched memory B cells** | **SIgA** | **IgA PC** | **SIgM** | **IgM PC** | **pre IgM anti-PC23** | **post IgM anti-PC23** | **pre IgA anti-PC23** | **post IgA anti-PC23** | **Pneumonia** | **Bronchiectasis** |
| --- | --- | --- | --- | --- | --- | --- | --- | --- | --- | --- | --- | --- | --- | --- | --- | --- | --- | --- | --- |
|  |  |  | **(mg/dl)** | **(mg/dl)** | **(mg/dl)** | **(mg/dl)** | **(mm/3)** | **(mm/3)** | **(mm/3)** |  |  |  |  | **(U/ml)** | **(U/ml)** | **(U/ml)** | **(U/ml)** |  |  |
| **GROUP 2** | **27** | 31 | 259 | 673 | 17 | 0 | 231 | 97 | 7 | - | - | - | - | 9.0 | 7.8 | 3.9 | 3.9 | No | No |
|  | **12** | 63 | 220 | 748 | 9 | 0 | 294 | 135 | 0 | - | - | - | - | 0.0 | 0.0 | 0.0 | 0.0 | Yes | Yes |
|  | **8** | 71 | 450 | 588 | 42 | 0 | 336 | 158 | 17 | - | - | - | - | 1.0 | 1.2 | 3.0 | 0.0 | No | No |
|  | **7** | 67 | 320 | 510 | 41 | 7 | 146 | 35 | 6 | + | + | - | + | nd | nd | nd | nd | No | Yes |
|  | **11** | 68 | 120 | 567 | 38 | 26 | 230 | 25 | 5 | + | + | - | + | 22.7 | 24.7 | 7.6 | 8.8 | No | Yes |
|  | **16** | 36 | 269 | 715 | 64 | 10 | 238 | 162 | 5 | + | + | + | ++ | nd | nd | nd | nd | No | No |
|  | **19** | 38 | 355 | 666 | 34 | 207 | 393 | 63 | 43 | + | + | - | + | nd | nd | nd | nd | No | No |
|  | **22** | 54 | 345 | 580 | 41 | 14 | 678 | 319 | 27 | ++ | ++ | - | + | nd | nd | nd | nd | No | No |
|  | **30** | 42 | 85 | 658 | 10 | 0 | 184 | 28 | 2 | + | + | + | ++ | 8.1 | 25.6 | 0.0 | 3.7 | No | No |
|  | **32** | 36 | 459 | 789 | 6 | 6 | 227 | 20 | 20 | + | + | - | - | 21.4 | 25.8 | 12.0 | 450.0 | No | No |
|  | **33** | 59 | 259 | 799 | 4 | 13 | 737 | 317 | 81 | + | + | - | - | 0.0 | 0.3 | 3.2 | 200.0 | No | No |
|  |  |  |  |  |  |  |  |  |  |  |  |  |  |  |  |  |  |  |  |
|  | **Mean** | 48.2 | 267.7 | 625.7 | 30.8 | 23.1 | 310.6 | 105.4 | 16.8 |  |  |  |  | 8.3 | 10.2 | 3.9 | 74.4 |  |  |
|  | **SD** | 15.0 | 118.4 | 94.6 | 19.6 | 60.7 | 196.4 | 109.6 | 24.2 |  |  |  |  | 9.7 | 12.6 | 4.3 | 172.8 |  |  |
|  | **n. (%)** |  |  |  |  |  |  |  |  | 8 (73%) | 8 (73%) | 2 (18%) | 6 (54%) |  |  |  |  | 1 (9%) | 3 (27%) |
|  |  |  |  |  |  |  |  |  |  |  |  |  |  |  |  |  |  |  |  |
| **HD** | **Mean** |  |  |  |  |  | 348 | 55 | 58 | +++ | + | +++ | ++ | 35.1 | 712.2 | 46.7 | 508.6 |  |  |
|  | **SD** |  |  |  |  |  | 155 | 35 | 37 |  |  |  |  | 56.2 | 363.1 | 62.4 | 305.7 |  |  |
|  | **Range** |  | 600-1600 |  | 40-250 | 70-315 |  |  |  |  |  |  |  |  |  |  |  |  |  |
