# Supplementary figures and images for: Lack of Gut Secretory Immunoglobulin A in Memory B-Cell Dysfunction-Associated Disorders: A Possible Gut-Spleen Axis
Source: Front Immunol. 2020 Jan 8;10:2937. doi: 10.3389/fimmu.2019.02937 (PMC6960143; doi:10.3389/fimmu.2019.02937)

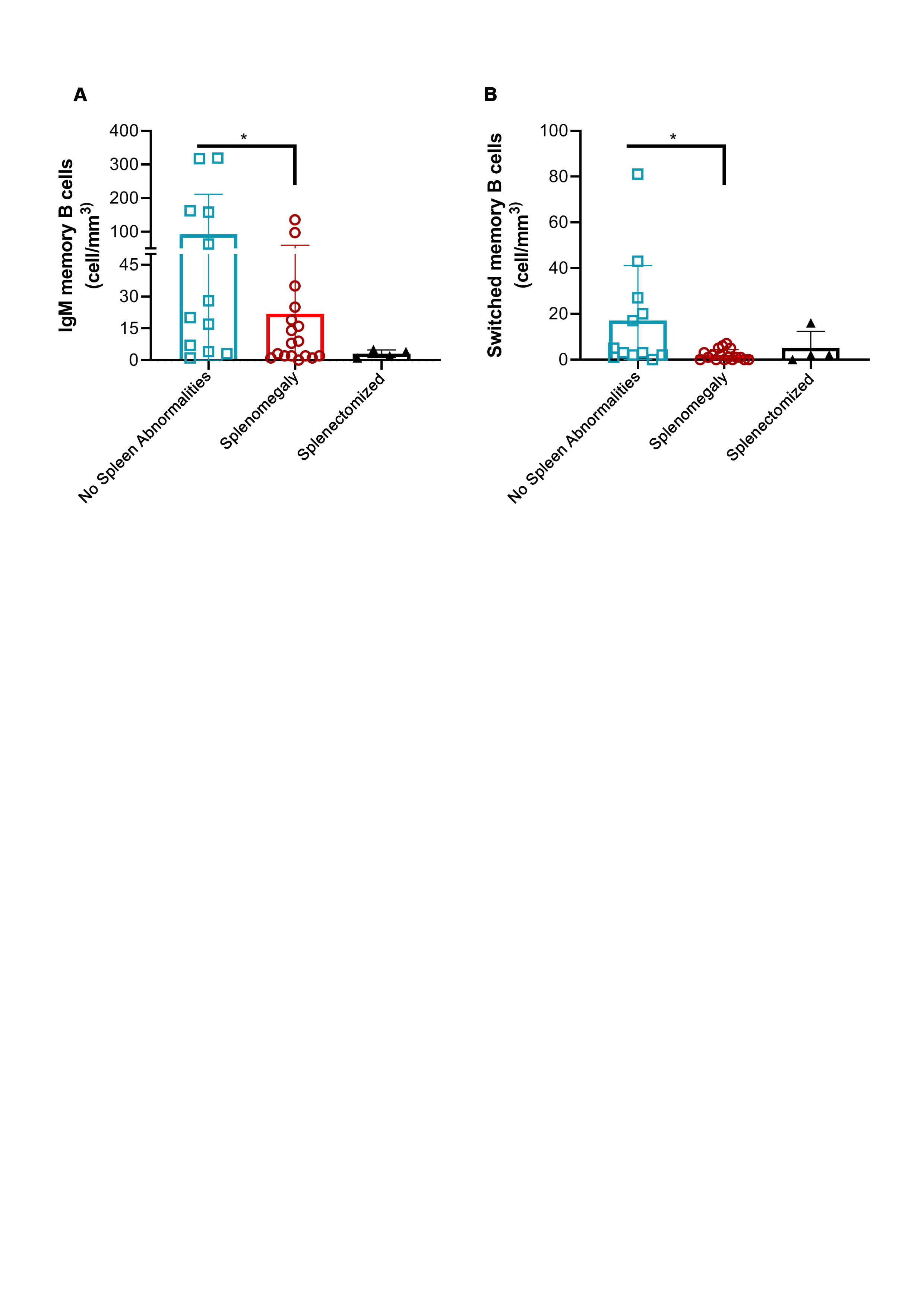

Supplement: Supplementary file 2 [file Image_1.JPEG]

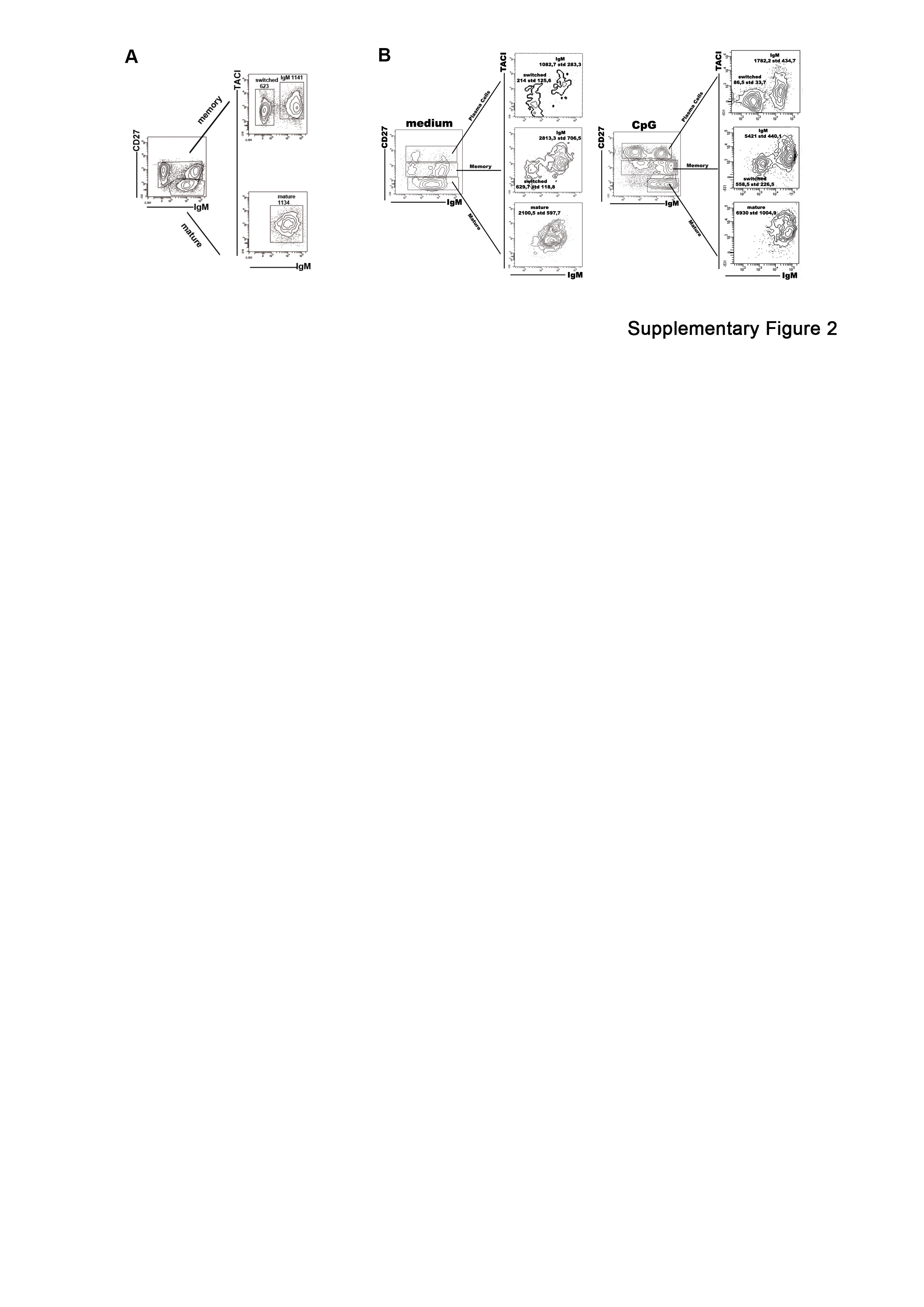

Supplement: Supplementary file 3 [file Image_2.JPEG]

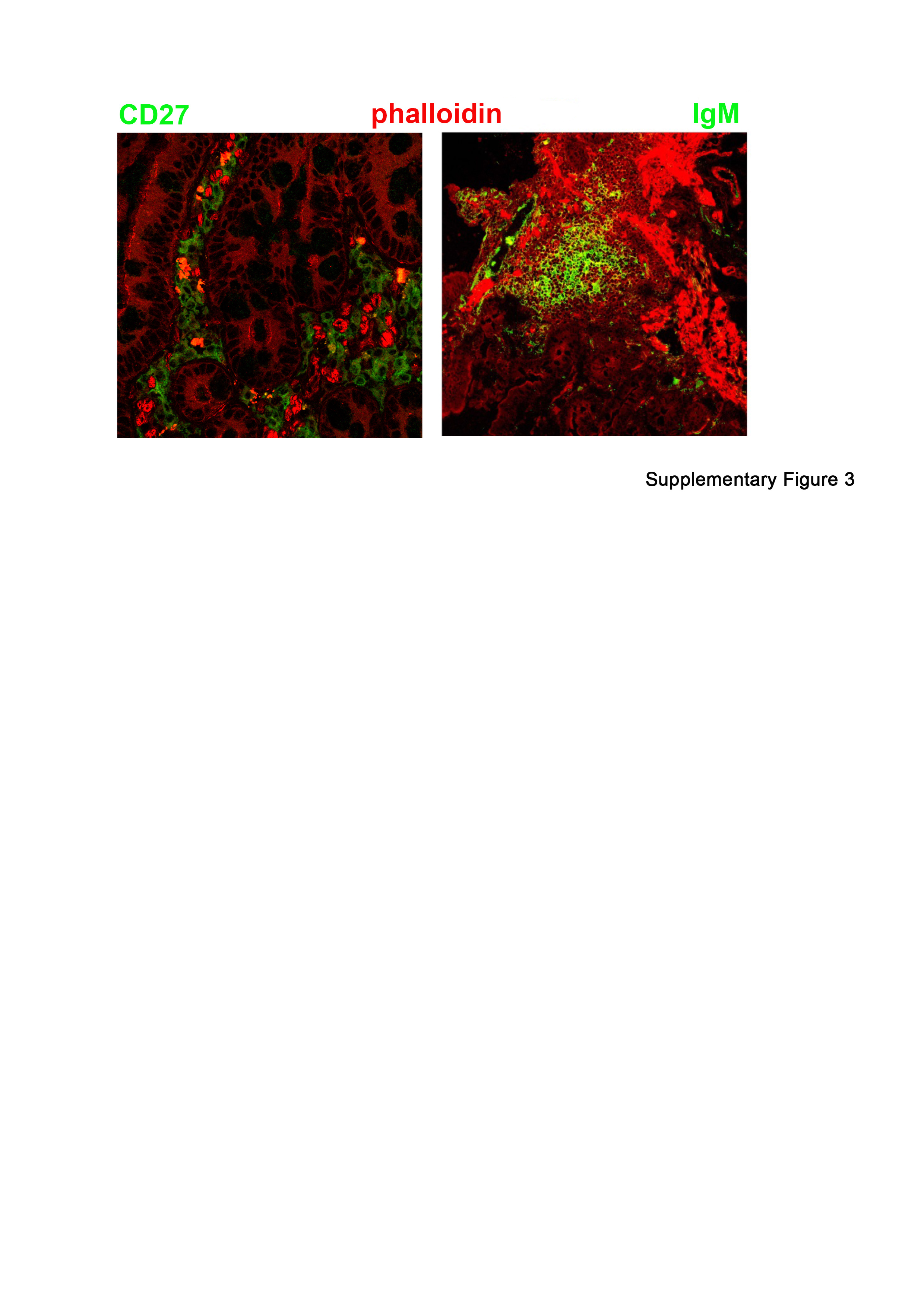

Supplement: Supplementary file 4 [file Image_3.JPEG]
